# Thrombectomy of Mild Stroke: An Analysis of the Nationwide Registry of the German Society for Neuroradiology

**DOI:** 10.1007/s00062-023-01262-6

**Published:** 2023-02-06

**Authors:** Ludger Feyen, Marcus Katoh, Patrick Haage, Nico Münnich, Martin Weinzierl, Christian Blockhaus, Stefan Rohde, Helge C. Kniep

**Affiliations:** 1 Department of Diagnostic and Interventional Radiology, Helios Klinikum Krefeld, Lutherplatz 40, 47805 Krefeld, Germany; 2 Faculty of Health, School of Medicine, University Witten/Herdecke, Alfred-Herrhausen-Str. 50, 58448 Witten, Germany; 3 Department of Diagnostic and Interventional Radiology, Helios Klinikum Wuppertal, Heusnerstr. 40, 42283 Wuppertal, Germany; 4 Department of Radiology and Neuroradiology, Klinikum Dortmund, Beurhausstr. 40, 44137 Dortmund, Germany; 5 Department of Neurosurgery, Helios Klinikum Krefeld, Lutherplatz 40, 47805 Krefeld, Germany; 6 Heart Centre Niederrhein, Department of Cardiology, Helios Clinic Krefeld, Krefeld, Germany; 7 Department of Radiology and Neuroradiology, Klinikum Dortmund, Beurhausstr. 40, 44137 Dortmund, Germany; 8 Department of Diagnostic and Interventional Neuroradiology, University Medical Center Hamburg-Eppendorf, Hamburg, Germany

**Keywords:** Stroke, Distal vessel occlusion, Posterior circulation, Anterior circulation, Reperfusion

## Abstract

**Purpose:**

Whether patients presenting with mild stroke (NIHSS at admission < 6) should be treated with mechanical thrombectomy (MT) is the subject of an ongoing debate. This retrospective study based on large-scale clinical data aims to identify factors associated with favorable outcome (FO) in patients with mild stroke.

**Methods:**

A total of 761 patients with mild stroke enrolled between 1 January 2020 and 31 December 2020 in the Quality Registry of the German Society for Neuroradiology were analyzed. The FO was defined as stable or improved NIHSS at discharge vs. admission. Descriptive statistics and multivariable logistic regression analyses were performed to identify factors associated with FO. Furthermore, a subgroup analysis of mild stroke based on distal vessel occlusion was conducted.

**Results:**

In this study 610 patients had FO with a median NIHSS at discharge of 1 (interquartile range, IQR, 0-2) and 151 had an unfavorable outcome (UO) with median NIHSS at discharge of 10 (IQR 13). Patients with FO had a slightly higher NIHSS at admission (4 vs. 3, *p* < 0.001), lower mTICI 0 (2.7% vs. 14.2%, *p* < 0.001), higher mTICI 3 (61.3% vs. 34.5%, *p* < 0.001) and a lower number of passes (1 vs. 2, *p* < 0.001). No statistically significant difference was observed for MT-related adverse events. Multivariable logistic regression suggested that NIHSS at admission (adjusted odds ratio (aOR) = 1.28, 95% confidence interval (CI) = 1.10–1.48), mTICI 2b (aOR = 5.44, CI = 2.06–15.03), mTICI 2c (aOR = 10.81, CI = 3.65–34.07) and mTICI 3 (aOR = 11.56, CI = 4.49–31.10) as well as number of passes (aOR 0.76, CI = 0.66–0.88) were significantly associated with FO. No MT-related adverse events were observed for distal vessel occlusions.

**Conclusion:**

The FO in patients with mild stroke undergoing MT was associated with successful recanalization. No significant differences between patients with FO and UO were found for MT-related adverse events, suggesting that MT complications have no significant effects on the outcome of these patients. MT might improve the prognosis also in patients with mild stroke based on distal vessel occlusions without significantly increasing the risk of adverse events.

## Introduction

Mechanical thrombectomy has become the standard of care for acute large vessel occlusions of the anterior circulation within a time window of 6 h after onset of symptoms [1]. Due to narrow inclusion criteria, several subgroups of patients with acute ischemic stroke potentially eligible for thrombectomy were excluded in the underlying studies [2–5] and were therefore not treated with mechanical thrombectomy in clinical practice according to current guidelines; however, also these patients with distal vessel occlusions or initial mild symptoms might benefit from mechanical thrombectomy [6].

Clinical experience confirms that even a mild stroke can significantly impact functional independence. The loss of the speech function or the loss of the motor function of the dominant arm for example constitute severe invaliding impairments despite the formally low corresponding NIHSS score of 3–4. A large earlier study and a recent meta-analysis reported similar outcomes of mechanical thrombectomy and best medical treatment for patients with mild stroke defined as NIHSS at admission < 6 [7, 8]. In line with these results, current AHA guidelines recommend that although benefit is uncertain, mechanical thrombectomy might be reasonable for patients with acute ischemic stroke, NIHSS at admission < 6 and causative occlusion of the internal carotid artery or proximal middle cerebral artery [9].

For distal occlusions, a subgroup analysis of patients with M2 occlusions from the HERMES study group reported a higher percentage of functional independence in patients treated with thrombectomy [10] however, these results are currently not reflected in the relevant guidelines [9, 11]. For anterior cerebral artery and posterior cerebral artery occlusions, previous studies indicated that thrombectomy may be effective but is associated with a higher rate of complications [12–16]. Consequently, current recommendations for distal occlusions are similarly cautious as for mechanical thrombectomy in mild stroke [9].

The aim of this retrospective study based on a large multicenter dataset was to investigate whether mechanical thrombectomy in patients with mild stroke defined by an initial NIHSS < 6 increases the probability of a favorable outcome and to identify factors that are associated with an improvement or worsening of symptoms defined as NIHSS decrease or increase from admission to discharge. Furthermore, a subgroup analysis of patients with mild stroke caused by distal vessel occlusions as defined by an exclusive occlusion of the M2 segment of the middle cerebral artery, posterior cerebral artery or anterior cerebral artery was performed. We hypothesized that successful mechanical thrombectomy in mild stroke is associated with improved patient outcome.

## Methods

### Study Sample and Data Collection

This retrospective study is based on the nationwide quality registry of the German Society for Neuroradiology (DGNR) and of the German Society for Interventional Radiology and Minimally Invasive Therapy (DEGIR). A total of 14,959 patients with acute vessel occlusion treated with thrombectomy were enrolled in the registry between 01/2020 and 12/2020. Inclusion criteria of our study were: NIHSS at admission < 6, available information on sex, age, i.v. thrombolysis, location of vessel occlusion, complications, time from groin puncture to recanalization, number of passes, recanalization result, NIHSS and mRS at discharge. Adverse events were defined as MT-related complications, further divided into dissections, subarachnoid hemorrhage, intraparenchymal hemorrhage and embolization into new territories. Methods of recanalization were selected according to the discretion of the treating interventionalist or neurologist. Intravenous thrombolysis was administered if the patient was eligible according to the national guidelines based on the decision of the treating neurologist. Neither approval of the institutional review board nor patient informed consent were required according to the local ethics committee due to the retrospective character of the analysis of anonymized patient records and imaging. All study protocols and procedures were conducted in accordance with the Declaration of Helsinki. The deidentified data can be requested from the corresponding author upon reasonable request after consultation with the professional society which provided the data.

### Definitions

Mild stroke was defined by NIHSS at admission < 6. Functional outcome was evaluated using the NIHSS at discharge. Favorable outcome was defined as NIHSS at discharge equal to or smaller than NIHSS at admission. Vice versa, unfavorable outcome was defined as NIHSS at discharge larger than NIHSS at admission. Distal vessel occlusions were defined as an exclusive occlusion of the M2 segment of the middle cerebral artery, posterior cerebral artery or anterior cerebral artery. The grade of reperfusion was determined using the mTICI scale [17].

### Statistical Analysis

Univariate comparisons were made using standard statistical measures (Fisher’s exact test for categorical variables, Mann-Whitney U test for non-normally continuous or ordinally scaled variables). Association of favorable outcome with other parameters was assessed using multivariable logistic regression adjusting for the following prespecified variables: age, sex, NIHSS on admission, administration of i.v. lysis, the presence of wake-up stroke, number of passes, achieved recanalization result and the occurrence of complications. The level of statistical significance was set to *p* < 0.05.

## Results

### Study Population

Out of the 14,959 patients treated with thrombectomy in Germany in 2020, 1531 patients with NIHSS < 6 at admission were identified. The NIHSS at discharge was missing in 655 patients and the mRS at discharge was missing in 115 patients so that 761 patients were included in the final analysis. Mean age was 74.5 years and median age was 77.5 years (IQR 17.1 years). Complications occurred in 8.5% of the patients, partly in combination. Parenchymal hemorrhage was observed in 1.5% of the patients, subarachnoid hemorrhage in 5.9%, embolization to a new vascular territory in 1.7% and dissections in 1.4% of the patients. Median NIHSS at discharge was 1 (IQR 3), median mRS at discharge was 1 (IQR 3) and 29 patients died in the hospital. No treatment-associated complications were noted in the deceased patients and no patient with a distal vessel occlusion died.

Of the patients 151 had an unfavorable outcome and 610 patients had a favorable outcome. Statistically significant differences between these groups were found for NIHSS at admission (favorable outcome: median 4, IQR 3 and unfavorable outcome: median 3, IQR 3, *p* < 0.001), wake-up stroke (favorable outcome: 36.6%, unfavorable outcome: 45.7%, *p* = 0.04), i.v. thrombolysis (favorable outcome: 41.8%, unfavorable outcome: 29.2%, *p* = 0.013), number of passes (favorable outcome: 1, IQR 1, unfavorable outcome: 2, IQR 3, *p* < 0.001) and recanalization results mTICI 0 (favorable outcome: 2.7%, unfavorable outcome: 14.2%, *p* < 0.001), mTICI 1 (favorable outcome: 0.7%, unfavorable outcome: 4%, *p* = 0.005), mTICI 2a (favorable outcome: 2.2%, unfavorable outcome: 8.1%, *p* = 0.0011), mTICI 2b (favorable outcome: 19.4%, unfavorable outcome: 29%, *p* = 0.014) and mTICI 3 (favorable outcome: 61.3%, unfavorable outcome: 34.5%, *p* < 0.001). No difference was observed in thrombectomy-related adverse events (Table 1).

**Table 1:** Patients with mild stroke as defined by an initial NIHSS < 6. Comparison of the patient group that deteriorated as defined by a worsening of the NIHSS at discharge compared to the initial NIHSS and the patient group with equal or improved NIHSS at discharge

	Unfavorable outcome	Favorable outcome	P‑value
No. of cases	151	610	–
Age, years, median (IQR)	78.5 (16.5)	77 (17.3)	0.7
Sex, % female	58.9	50.8	0.08
NIHSS, median (IQR)	3 (3)	4 (3)	< 0.001**
Wake-up stroke, %	45.7	36.6	0.04*
Occlusion site, %			
*ACA*	5.7	4.8	0.67
*BA*	4.9	8.9	0.13
*Carotid cavernous*	4.2	5.3	0.68
*Cervical carotid*	7.0	5.3	0.43
*Intradural carotid*	16.3	15.8	1
*Carotid bulb*	10.6	9.4	0.75
*M1*	24.1	23.2	1
*M1 distal*	7.8	8.5	0.87
*M2*	17.7	12.8	0.18
*PCA*	1.4	3.4	0.28
*V4*	0	2.7	0.05
I.v. thrombolysis, %	29.2	41.8	0.01
Time from groin puncture to recanalization, median, minutes (IQR)	48 (46)	44 (48.8)	0.36
No. of passes, median	2 (3)	1 (1)	< 0.001**
Stent retriever, %	68.9	74.9	0.15
mTICI, %			
*0*	14.2	2.7	< 0.001**
*1*	4	0.7	0.005*
*2a*	8.1	2.2	0.001*
*2b*	29	19.4	0.01*
*2c*	10.1	13.8	0.28
*3*	34.5	61.3	< 0.001**
Complications *n*, %	10 (6.6)	55 (9)	0.42
*Embolization in new* *territory n, %*	0	13 (2.1)	0.083
*Parenchymal hemorrhage* *n, (%)*	4 (2.6)	7 (1.1)	0.24
*SAH n, (%)*	9 (6)	36 (5.9)	1
*Dissection n, (%)*	2 (1.3)	8 (1.3)	1
mRS discharge, median (IQR)	4 (2)	1 (2)	< 0.001**
NIHSS discharge, median (IQR)	10 (13)	1 (2)	< 0.001**
Death, %	19.2	–	–

*ACA* anterior cerebral artery, *PCA* posterior cerebral artery, *M1* M1 segment of the middle cerebral artery, *M2* M2 segment of the middle cerebral artery, *SAH* subarachnoid hemorrhage, *BA* basilar artery, *V4* V4 segment of the vertebral artery

* level of significance *p* < 0.05, ** level of significance *p* < 0.001

Of the patients 61 suffered from mild stroke based on distal vessel occlusions defined as occlusion of the M2 segment of the middle cerebral artery, the anterior cerebral artery or the posterior cerebral artery. Of these patients, 11 had an unfavorable outcome and 50 patients had a favorable outcome. Differences between these groups were found for sex (favorable outcome: 48% female, unfavorable outcome: 90.9% female, *p* = 0.016), recanalization results mTICI 2a (favorable outcome: 2%, unfavorable outcome: 27.3%, *p* = 0.017) and mTICI 3 (favorable outcome: 73.5%, unfavorable outcome: 27.3%, *p* = 0.01) (Table 2).

**Table 2:** Patients with mild stroke as defined by an initial NIHSS < 6 based on distal vessel occlusions as defined by the M2 segment of the middle cerebral artery, the posterior cerebral artery and the anterior cerebral artery. Comparison of the patient groups with favorable and unfavorable outcome

	Unfavorable outcome	Favorable outcome	P‑value
No. of cases	11	50	–
Age, years, median (IQR)	78.8 (18.6)	79.4 (17.6)	0.66
Sex, % female	90.9	48	0.016*
NIHSS, median (IQR)	4 (1.5)	4 (2)	0.63
Wake-up stroke, %	27.3	34	1
Occlusion site, %			
*ACA*	9.1	12	1
*M2*	81.8	68	0.48
*PCA*	9.1	22	0.67
I.v. thrombolysis, %	36.4	34.9	1
Time from groin puncture to recanalization, median, minutes (IQR)	45 (46.5)	40 (46.5)	0.54
No. of passes, median	3 (3.5)	1 (2)	0.09
Stent retriever, %	100	100	–
mTICI, %			
*0*	18.2	2	0.08*
*1*	9.1	4.1	0.46
*2a*	27.3	2	0.017
*2b*	18.2	14.3	0.66
*2c*	0	4.1	1
*3*	27.3	73.5	0.01*
Complication	0	0	0
*Embolization in new* *territory, %*	0	0	0
*SAH, %*	0	0	0
*Parenchymal* *hemorrhage*	0	0	0
mRS discharge, median (IQR)	4 (1)	1 (2)	< 0.001**
NIHSS discharge, median (IQR)	10 (6)	1 (2)	< 0.001**

*ACA* anterior cerebral artery, *PCA* posterior cerebral artery, *M2* M2 segment of the middle cerebral artery, *SAH* subarachnoid hemorrhage

*: level of significance *p* < 0.05, **: level of significance *p* < 0.001

### Multivariable Regression Analysis

**Fig. 1:**
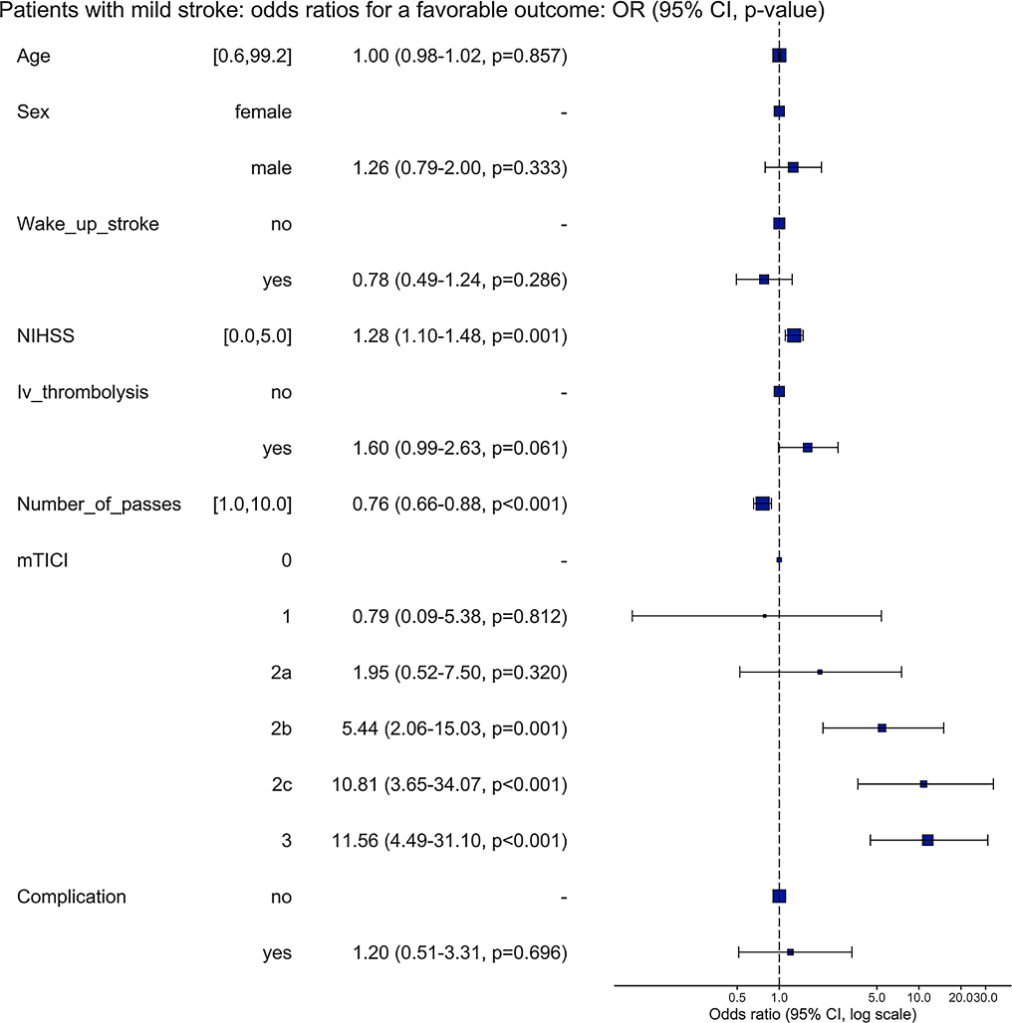
Odds ratio plot for patients with mild stroke. Depicted are the adjusted odds ratios, the 95% confidence intervals and the respective *p*-values for the variables age, sex, treatment with i.v. lysis, NIHSS at admission, achieved recanalization grade as measured by the mTICI score, the presence of wake-up stroke, number of passes and total complications arising during treatment

Figure 1 shows the results from the multivariable regression analysis for patients with favorable and unfavorable outcome. The adjusted odds ratio (aOR) for a favorable outcome was 1.28 (CI 1.10–1.48, *p* = 0.001) for NIHSS at admission, 1.60 (CI 0.99–2.63, *p* = 0.061) for i.v. thrombolysis, 5.44 (CI 2.06–15.03, *p* = 0.001) for mTICI 2b, 10.81 (CI 3.65–34.07, *p* < 0.001) for mTICI 2c and 11.56 (CI 4.49–31.10, *p* < 0.001) for mTICI 3, 0.78 (CI 0.49–1.24, *p* = 0.286) for wake-up stroke and 0.76 (CI 0.66–0.88, *p* < 0.001) for number of passes. In the additional subgroup analysis for patients with mild stroke based on distal occlusions no analyzed factor reached the level of significance of *p* < 0.05.

## Discussion

This study based on 761 patients confirms that successful recanalization with mechanical thrombectomy is associated with a favorable outcome in patients with mild stroke. No significant differences between favorable and unfavorable outcome groups were found for mechanical thrombectomy-related adverse events in patients with mild stroke and in the subgroup of patients with mild stroke due to distal vessel occlusions, suggesting that mechanical thrombectomy-related complications had no significant effects on the outcome in these patients.

Occlusions causative for mild stroke were localized in different vascular locations in the anterior and posterior circulation; however, no significant difference was found between the locations of the stroke-causing vessel occlusion for patients with favorable vs. unfavorable outcome.

Key pillar of the treatment of ischemic stroke is the reopening of the causative vascular occlusion as fast as possible. Significantly better functional outcomes were reported for patients with vessel occlusions of the proximal anterior circulation and the basilar artery that were treated with mechanical thrombectomy than with treatment in a stroke unit without thrombectomy or i.v. thrombolysis alone [1, 18–22]. Our analysis shows that patients with good outcome have a significantly higher rate of mTICI 3 compared to patients with unfavorable outcome, suggesting that full recanalization might be an important factor for good outcome in mild stroke.

A previous study reported higher rates of symptomatic intracerebral hemorrhage of patients with mild stroke who were treated with thrombectomy compared to patients who were treated solely with i.v. lysis with no improvement of excellent and independent functional outcomes in the thrombectomy group [8]; however, this study included patients that were treated between 2012 and 2017. We analyzed data from the year 2020 of patients treated with state-of-the-art devices and did not find a significant association between thrombectomy-related complications and functional outcome. Most notably, there was no significant association between parenchymal hemorrhage and clinical deterioration.

Overall, the strongest association was found between successful endovascular recanalization and favorable outcome. Our study confirms the results of smaller previous studies reporting that successful thrombectomy in patients with initially mild symptoms leads to a significantly better outcome [23, 24].

For distal vessel occlusions, mechanical thrombectomy is particularly controversial. Distal vessel occlusions generally show a more favorable clinical course [6]. The alternative treatment with i.v. thrombolysis showed higher recanalization rates of about 50% in the peripheral vessel compared to proximal vessel occlusions with reopening rates of between 13% for the basilar artery and 35% for the M1 segment of the middle cerebral artery [18]. Previous studies showed good recanalization rates of up to 70% in the peripheral anterior circulation with increased complication rates for endovascular procedures [13–15]. A previous multicenter study concluded that recanalization of isolated occlusions of the posterior cerebral artery is feasible and effective with a reported recanalization rate of 86%, but further studies to investigate the safety of the method are needed [16]. The superior reopening rates of thrombectomy in the vessel periphery in initial studies must therefore be weighed individually against rare but potentially fatal complications. In the present study, no complications in the relatively small patient group of mild stroke due to distal vessel occlusions occurred. The strongest correlation in this patient subgroup was found between successful endovascular recanalization and favorable outcome, suggesting that endovascular recanalization might also be safe and efficient in patients with mild stroke due to distal vessel occlusions. Notably, the percentage of females was significantly higher in patients with unfavorable outcome. These findings corroborate results from other studies reporting worse functional outcomes for females after ischemic stroke [25]; however, limited sample size of the subgroup analysis might reduce generalizability of results. Relevant insights can be expected from the currently enrolling RCTs evaluation mechanical thrombectomy for distal vessel occlusions (EndovaSCular TreAtment to imProve outcomEs for Medium Vessel Occlusions (ESCAPE-MEVO) and randomized controlled trial of the clinical outcome and safety of endovascular versus standard medical therapy for stroke with medium sized vessel occlusion (FRONTIER-AP)) [26, 27].

### Limitations

Complication rates and recanalization rates in the analyzed database are self-reported and not supervised as in randomized controlled trials. The reported complication rate in this database and the reported recanalization rates are, however, comparable to the recanalization rates of the EXTEND-IA and the SWIFT-Prime trials with 86% and 83%, respectively, and reported complications with 6–8% [28, 29]. We therefore assume sufficient quality of the analyzed data in analogy with an earlier publication [19]. The lack of long-term follow-up data, especially the mRS score at 90 days constitutes a limitation of our study; however, previous studies indicated that a NIHSS after the intervention might serve as a sufficient surrogate for long-term functional outcome after thrombectomy in daily clinical practice [30, 31]. In addition, mRS assessment is heavily weighted toward motor functions, which might be less relevant for patients with mild stroke. Due to the significantly more granular assessment of NIHSS vs. mRS, NIHSS might be better suited for the evaluation of functional impairments after mild stroke.

## Conclusion

Successful recanalization after mechanical thrombectomy with equal or better than mTICI 2b was associated with favorable outcome in patients with mild stroke defined as NIHSS at admission < 6. In patients with mild stroke due to distal vessel occlusions, patients with favorable outcome had a significantly higher percentage of full recanalization (mTICI 3). Results furthermore suggest that clinical deterioration was not associated with mechanical thrombectomy-related complications but with failed recanalization. Prospective studies that evaluate thrombectomy in mild stroke and distal vessel occlusions are required to further confirm results.
